# Stress myocardial blood flow correlates with ventricular function and synchrony better than myocardial perfusion reserve: A Nitrogen-13 ammonia PET study

**DOI:** 10.1007/s12350-016-0669-y

**Published:** 2016-09-28

**Authors:** Luis Eduardo Juárez-Orozco, Erick Alexanderson, Rudi A. Dierckx, Hendrikus H. Boersma, Johannes L. Hillege, Clark J. Zeebregts, Myriam M. Martínez-Aguilar, Antonio Jordán-Ríos, Ana Gabriela Ayala-German, Niek Prakken, Rene A. Tio, Riemer H. Slart

**Affiliations:** 1 Department of Nuclear Medicine and Molecular Imaging, University of Groningen, University Medical Center Groningen, Groningen, The Netherlands; 2 PET/CT Unit, Faculty of Medicine, National Autonomous University of Mexico, Mexico City, Mexico; 3 Department of Nuclear Cardiology, Instituto Nacional de Cardiología “Ignacio Chávez”, Juan Badiano #1 Col. Sección XVI Del. Tlalpan, C.P 14080 Mexico City, Mexico; 4 Department of Cardiology, University of Groningen, University Medical Center Groningen, Groningen, The Netherlands; 5 Department of Epidemiology, University of Groningen, University Medical Center Groningen, Groningen, The Netherlands; 6 Department of Surgery (Division of Vascular Surgery), University of Groningen, University Medical Center Groningen, Groningen, The Netherlands; 7 Department of Clinical Pharmacy and Pharmacology, University of Groningen, University Medical Center Groningen, Groningen, The Netherlands; 8 Mexican Faculty of Medicine, La Salle University, Mexico City, Mexico; 9 Department of Biomedical Photonic Imaging, University of Twente, Enschede, The Netherlands; 10 Department of Physiology, Faculty of Medicine, National Autonomous University of Mexico, Mexico City, Mexico

**Keywords:** PET, stress myocardial blood flow, myocardial perfusion reserve, ventricular function, coronary artery disease

## Abstract

**Background:**

Cardiac PET quantifies stress myocardial blood flow (MBF) and perfusion reserve (MPR), while ECG-gated datasets can measure components of ventricular function simultaneously. Stress MBF seems to outperform MPR in the detection of significant CAD. However, it is uncertain which perfusion measurement is more related to ventricular function. We hypothesized that stress MBF correlates with ventricular function better than MPR in patients studied for suspected myocardial ischemia.

**Methods:**

We studied 248 patients referred to a rest and adenosine-stress Nitrogen-13 ammonia PET. We performed a multivariate analysis using systolic function (left ventricular ejection fraction, LVEF), diastolic function (mean filling rate in diastole, MFR/3), and synchrony (Entropy) as the outcome variables, and stress MBF, MPR, and relevant covariates as the predictors. Secondarily, we repeated the analysis for the subgroup of patients with and without a previous myocardial infarction (MI).

**Results:**

166 male and 82 female patients (mean age 63 ± 11 and 67 ± 11 year, respectively) were included. 60% of the patients presented hypertension, 57% dyslipidemia, 21% type 2 diabetes mellitus, 45% smoking, and 34.7% a previous MI. Mean stress MBF was 1.99 ± 0.75 mL/g/min, MPR = 2.55 ± 0.89, LVEF = 61.6 ± 15%, MFR/3 = 1.12 ± 0.38 EDV/s, and Entropy = 45.6 ± 11.3%. There was a significant correlation between stress MBF (*P* < .001) and ventricular function. This was stronger than the one for MPR (*P* = .063). Sex, age, diabetes, and extent of previous MI were also significant predictors. Results were similar for the analyses of the 2 subgroups.

**Conclusion:**

Stress MBF is better correlated with ventricular function than MPR, as evaluated by Nitrogen-13 ammonia PET, independently from other relevant cardiovascular risk factors and clinical covariates. This relationship between coronary vasodilatory capacity and ventricular function is sustained across groups with and without a previous MI.

**Electronic supplementary material:**

The online version of this article (doi:10.1007/s12350-016-0669-y) contains supplementary material, which is available to authorized users.

## Introduction

Cardiac positron emission tomography (PET) imaging has developed as an important adjuvant for diagnostic, prognostic, and therapeutic evaluation in the evolving profile of coronary artery disease (CAD), and is currently the reference technique for the quantification of myocardial perfusion in absolute terms.^1^ This quantification of myocardial blood flow (MBF) is performed during rest and pharmacological stress, and the calculated ratio of stress MBF to rest MBF is known as the myocardial perfusion reserve (MPR). Stress MBF and MPR have shown superior diagnostic performance as compared to the evaluation of relative myocardial tracer retention and semiquantitative assessment.^2^–^7^

Recently, it has been suggested that stress MBF may perform better than MPR in the detection of flow-limiting CAD. This may be especially true in populations without previously known CAD or in conditions that show a higher rest MBF such as women, older patients, or those with arterial hypertension.^8^ Further, only a couple of studies have partially addressed the prognostic value of stress MBF over MPR for the occurrence of cardiovascular events.^9^,^10^

Furthermore, ECG-synchronized (gated) PET datasets can be simultaneously acquired offering the possibility to assess three areas of ventricular function, namely: systolic (i.e., ejection) and diastolic (i.e. filling) function,^11^–^13^ and ventricular synchrony (uniformity of left ventricular contraction).^14^,^15^ This is useful because cardiac function measurements can provide information for the evaluation of the effects of myocardial ischemia.^16^ Indeed, progressive hampering of ventricular function also constitutes an important component when addressing cardiovascular prognosis and risk stratification. As such, although quantitative measures of myocardial perfusion are predictive of coronary anatomic findings of CAD, it is still unknown whether stress MBF or MPR is better correlated with resulting ventricular function.

Therefore, the aim of the present study was to evaluate whether stress MBF or MPR, as measured by PET myocardial perfusion imaging, better correlates with ventricular function in the general population of patients with known or suspected CAD. Secondarily, we explored the relationship of stress MBF and MPR with ventricular function in patients with and without a previous myocardial infarction (MI) as it is known that there are differences in ventricular perfusion and function dynamics.

A supplementary analysis for subgroups of interest within patients without a previous MI (e.g. women and patients with arterial hypertension) was also conducted.

## Methods

### Population

A retrospective study was conducted including 248 patients referred to the PET/CT Unit at the National Autonomous University of Mexico in Mexico City by their cardiologist for the evaluation of suspected myocardial ischemia. Demographic and clinical data were retrieved through the electronic patient archive system. Several clinically relevant confounders were included in this study: sex, age, body mass index (BMI), hypertension, dyslipidemia, type 2 diabetes mellitus (DM), smoking habit, and semiquantitative perfusion scores (see ahead). Ethical approval was obtained for the conduction of the study from the local institutional ethics committee.

### PET Acquisition

PET images were acquired on a whole-body 64-slice PET/CT scanner (Biograph True Point; Siemens Medical Solutions). PET data were acquired in 3-D list mode. Patients were studied after an overnight fast, and all refrained from caffeine-containing beverages or theophylline-containing medications for 24 hours before the study. Myocardial perfusion was assessed at rest and during vasodilator stress with adenosine and Nitrogen-13 ammonia as the perfusion radiotracer. Two CT-based transmission scans (120 kVp; 20-30 mA; helical scan mode with a pitch of 1.35) were obtained before the rest perfusion studies and after the stress perfusion studies with normal breathing for correction of photon attenuation for PET. The registration of the CT attenuation map with the PET images was verified visually by an experienced technologist and alignment was corrected if necessary by manual 3-D translation. Regional myocardial perfusion was first assessed during rest using 740 MBq of Nitrogen-13 ammonia. Rest imaging extended for 10 minutes and began a few seconds before the injection. The radiotracer was administered as a single peripheral intravenous bolus (3-5 seconds) followed by a 10-mL saline flush. Thirty minutes later, a pharmacological stress test was performed, beginning with the injection of adenosine during a 6-minute period (140 mg/kg per minute). A second dose of 740 MBq of Nitrogen-13 ammonia was injected at the third minute of the adenosine infusion. Stress images acquisition was started a few seconds before the radiotracer injection. Sixteen dynamic frames were reconstructed (twelve 10-s, two 30-s, one 1-minute, and one 6-minute frames, for a total of 10 minutes). Standard reconstruction (2-D attenuation-weighted OSEM) was used with 3 iterations and 14 subsets and 3-D postfiltering with a 5-mm Gaussian kernel filter. Transverse data were reformatted to a 168×168×47 matrix with 2 mm pixels for each dynamic frame.

### Perfusion Data Analysis

#### Semiquantitative Myocardial Perfusion

Images were interpreted semiquantitatively using the standard American Heart Association 5-point scoring system 17 and traditional metrics were documented: summed difference score (SDS), summed stress score (SSS), and summed rest score (SRS). SRS (in a fixed perfusion defect) was considered as a measure of the extent and severity of a previous MI.

#### Quantitative Myocardial Perfusion

Left ventricular contours and input function regions were obtained automatically allowing minimal observer intervention in QPS software package (Cedars-Sinai, Los Angeles, CA, USA). Dynamic myocardial samples were obtained from the polar map by analyzing all time frames within the fixed left ventricular contour boundaries. Quantitative rest and stress MBF values (mL/g/minute) were computed for each sample on the polar map as described previously^18^ using a previously described 2-tissue compartment pharmacokinetic model for Nitrogen-13 ammonia.^19^ MPR was calculated as the ratio between stress MBF and rest MBF (making it a unitless variable). Rest MBF was corrected accordingly for the rate-pressure product (RPP).^20^ The global MPR and stress MBF were calculated within the whole left ventricular region (as defined by the left ventricle long-axis plane) as parameters of interest for our analysis.

### Ventricular Function Data Analysis

ECG-gated stress images were analyzed with the QGS software package (Cedars-Sinai, Los Angeles, CA, USA).^21^ Short-axis images were processed and ventricular edges and cavity volumes were calculated for each of the re-binned 8 dynamic frames that were reconstructed for the average cardiac cycle. The algorithm for determining edges and calculating volume has been described previously.^22^

Systolic function was evaluated through left ventricular ejection fraction (LVEF) (% of the end-diastolic volume ejected) using the average time-volume curves. Next, diastolic function was evaluated with the mean filling rate during the first third of diastole (MFR/3) as a surrogate marker. MFR/3 was obtained from the first derivative of the left ventricular time-volume curve and expressed in end-diastolic volumes per second (EDV/s) as it is adjusted to the ventricle dimensions on a patient-by-patient basis.^23^,^24^

Finally, ventricular synchrony was evaluated through Entropy, which is a measurement of uniformity of the onset and progression of wall motion throughout the cardiac cycle.^25^ Entropy was expressed as a percentage (ranging from 0 to 100) with greater percentages reflecting a lesser uniformity of ventricular fiber contraction.^14^ It was obtained by phase analysis (embedded in the QGS software package [Research Edition, PET Processing plugin, Cedars-Sinai]). Entropy was used because it constitutes an expression of (dys)synchrony that is not dependent on phase similarity^25^ as other described parameters (Bandwidth and Standard Deviation).

### Statistical Analysis

All continuous variables are described as mean ± standard deviation, and categorical variables are expressed as frequencies with percentages. Between-group comparisons were made using independent samples *t* tests.

The biserial correlations (using Pearson’s correlation coefficient) between LVEF, MFR/3, and Entropy were evaluated. Next, a general multivariate analysis of covariance (MANCOVA) was performed including sex, age, BMI, hypertension, dyslipidemia, type 2 DM, smoking habit, SRS, SSS, stress MBF, and MPR in the model as the independent (i.e. predictive) variables, and LVEF, MFR/3, and Entropy as the dependent (i.e. outcome) variables. The independent significance of the included predictors was tested using Pillai’s trace criterion with an approximate *F* statistic. Further, the multivariate analyses were repeated including the same independent and dependent variables for patients with and without imaging evidence of a previous MI. Additionally, the effect sizes for the predictors (η^2^) are reported and graphically depicted.

Finally, supplementary analyses were performed in both female and hypertensive patients without a previous MI.

All statistical analyses were performed with SPSS (Released 2013. IBM SPSS Statistics for Windows, Version 22.0. Armonk, NY: IBM Corp., USA). A *P*-value of <0.05 was considered statistically significant.

## Results

**Figure 1:**
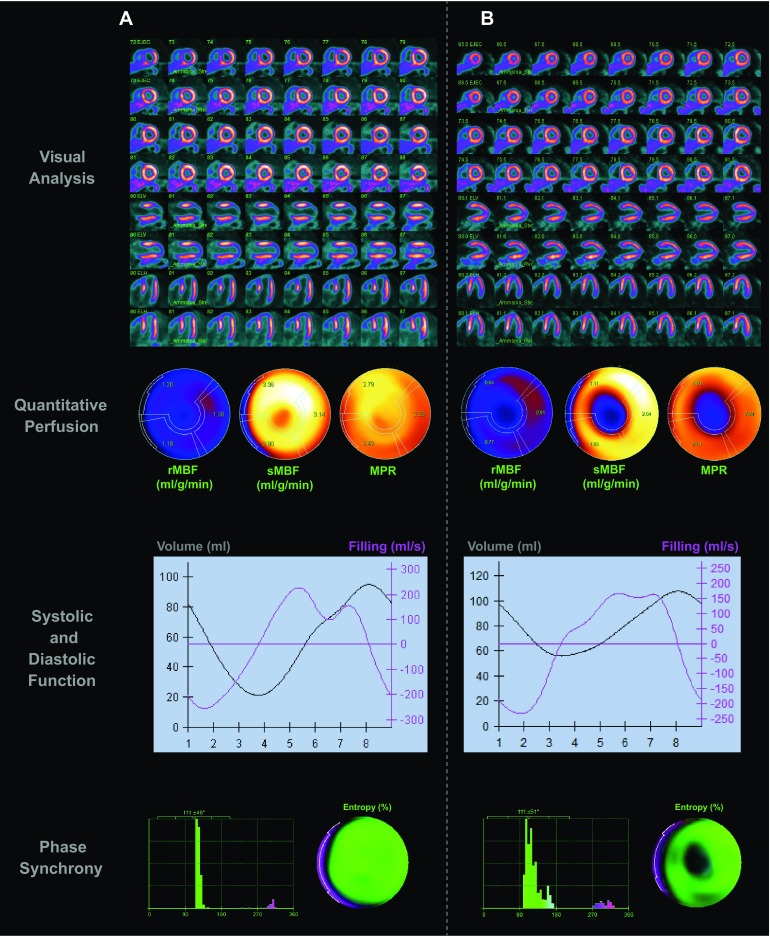
Representative cases from the spectrum of patients in the study. **A** shows the perfusion and functional results of a 55-year-old female with a low-likelihood cardiovascular risk, no quantitative perfusion abnormalities: MPR = 2.58, sMBF = 2.22 mL/g/min, and preserved systolic, diastolic, and synchronic function: LVEF = 75%, MFR/3 = 1.25, E = 34%. **B** Shows a 64-year-old male with history of a previous distal anterior and apical MI with residual anteroapical ischemia: MPR = 1.88, sMBF = 1.43 and diminished systolic, diastolic, and synchronic function: LVEF 47%, MFR/3 = 0.55, E = 61%

**Figure 2:**
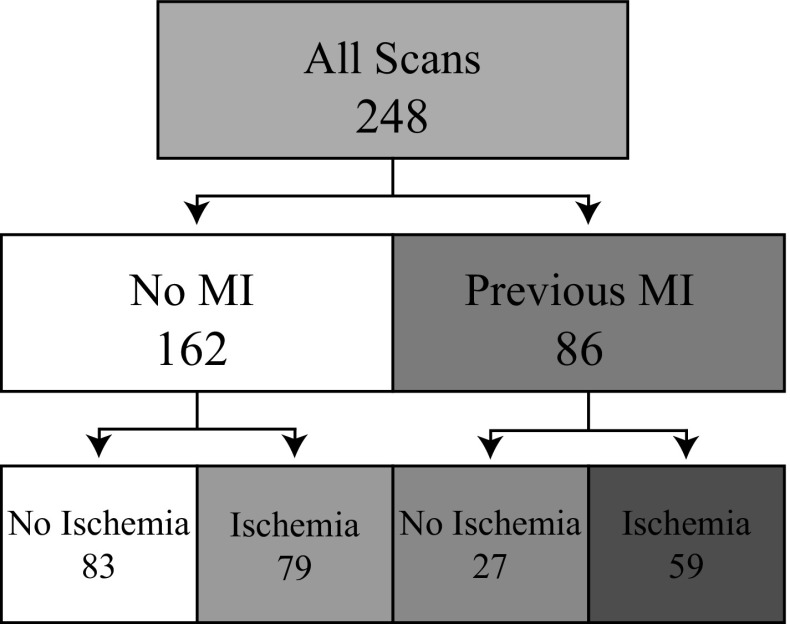
Division of scans according to their interpretation

Baseline characteristics, semiquantitative, quantitative perfusion, and ventricular function data are presented in Table 1. We found a high prevalence of HTN (60%) and dyslipidemia (57%) in the whole cohort, while nearly a quarter had undergone previous revascularization. When comparing patients with and without a previous MI, a significantly higher prevalence of type 2 DM, smokers, and dyspnea was documented in the MI group. The scan results of two representative patients are shown in Figure 1. Figure 2 shows the proportion of scans interpreted to have ischemia, MI, or both. A greater proportion of scans with a previous MI showed ischemic findings, while a smaller proportion of scans without evidence of a previous MI had ischemia.

**Table 1:** Baseline population characteristics

Variable	All n = 248	No previous MI n = 162	Previous MI n = 86	*P* value
Demographics—mean (SD)				
Age (years)	64 (11.2)	63.2 (11.3)	65.4 (10.8)	.137
Women/men (n)	82/166	65/97	17/69	.002
BMI (kg/m^2^)	27.8 (4.2)	28.0 (4.3)	27.4 (3.8)	.334
Risk factors—n (%)				
Arterial hypertension	149 (60)	101 (62)	48 (56)	.292
Dyslipidemia	141 (57)	90 (56)	51 (59)	.540
Type 2 DM	51 (21)	25 (15)	26 (30)	.007
Smokers	112 (45)	65 (40)	47 (55)	.021
Cardiovascular history—n (%)				
Asymptomatic	60 (24)	54 (33)	6 (7)	<.001
Angina	130 (52)	80 (49)	50 (58)	.178
Dyspnea	135 (54)	75 (46)	60 (70)	<.001
Previous revascularization	60 (24)	15 (9)	45 (52)	<.001
Semiquantitative perfusion metrics—mean (SD)				
SRS	4 (7)	1 (1)	10 (9)	<.001
SSS	9 (10)	5 (6)	17 (11)	<.001
SDS	5 (7)	5 (6)	7 ()	.001
Quantitative perfusion measurements—mean (SD)				
Rest MBF (mL/g/min)	0.84 (0.34)	0.87 (0.33)	0.79 (0.34)	.073
Stress MBF (mL/min/gr)	1.99 (0.75)	2.21 (0.73)	1.55 (0.58)	<.001
MPR	2.55 (0.89)	2.72 (0.89)	2.18 (0.79)	<.001
Ventricular function measurements—mean (SD)				
LVEF (systolic)	61.6 (15.1)	67.9 (9.7)	49.0 (15.9)	<.001
MFR/3 (diastolic)	1.12 (0.38)	1.21 (0.35)	0.91 (0.36)	<.001
Entropy (synchrony)	45.6 (11.3)	42.3 (8.7)	52.8 (12.4)	<.001

*BMI* body mass index; *DM* diabetes mellitus; *MI* myocardial infarction; *MBF* myocardial blood flow; *MPR* myocardial perfusion reserve

Differences were found in clinical characteristics, perfusion (semiquantitative scores, stress MBF, and MPR), and ventricular function parameters (LVEF, MFR/3, and Entropy) between patients with and without MI. There was a higher, although not significant, rest MBF in patients without MI.

The corresponding data for the subsample of women and patients with arterial hypertension can be found in Supplementary Material—Online Resource 1 and showed comparable distribution of baseline characteristics with the exception of a higher proportion of asymptomatic patients and a lower proportion of previous revascularization procedures. Also, there was a comparable rest and stress MBF in women and hypertensive patient groups.

### Biserial Correlations Between Dependent Variables

We documented significant and strong correlations between systolic, diastolic, and synchrony function variables. Pearson’s correlation coefficients showed significance between LVEF and MFR/3, LVEF and Entropy, and MFR/3 and Entropy. These results are depicted in the correlation matrix shown in Table 2.

**Table 2:** Biserial correlations between dependent variables analyzed through Pearson’s correlation coefficient

	LVEF	MFR/3	*E*
LVEF	1	0.612*	−0.698*
MFR/3	0.612*	1	−0.563*
*E*	−0.698*	−0.563*	1

*E* entropy; *LVEF* left ventricular ejection fraction; *MFR/3* mean filling rate during the first third of the diastole.

* *P* < 0.001

### Multivariate Analysis of Covariance

The multivariate analysis demonstrated that stress MBF is an independent significant predictor (*P* < .001, η^2^ = 0.111) of the ventricular function (LVEF, MFR/3, and E), while MPR showed only a trend toward significance (*P* = 0.063). From the other clinically relevant variables included in the model, sex (*P* = .003), age (*P* = .004), type 2 DM (*P* = .025), and SRS (*P* < .001, η^2^ = 0.284) were also found to be significant predictors (Table 3).

**Table 3:** Multivariate model results for significant predictors of the integrative ventricular function, n = 248

Multivariate analysis of covariance						
Dependent variables	Independent variables	Pillai’s Trace Value (and η^2^)	F	Hypothesis df	Error df	*P* value
LVEF MFR/3 Entropy	Intercept	0.630	98.705	3.0	174.0	<.001
	Sex	0.077	4.861			.003*
	Age	0.073	4.586			.004*
	Hypertension	0.015	0.870			.458
	Dyslipidemia	0.010	0.606			.612
	Type 2 DM	0.052	3.187			.025*
	Smoking	0.033	1.967			.121
	BMI	0.031	1.841			.141
	SRS	0.284	22.966			<.001*
	SSS	0.028	1.695			.170
	Stress MBF	0.111	7.253			<.001*
	MPR	0.041	2.474			.063

*BMI* body mass index; *df* degrees of freedom; *DM* diabetes mellitus; *LVEF* left ventricular ejection fraction; *MBF* myocardial blood flow; *MFR/3* mean filling rate during the first third of the diastole; *MPR* myocardial perfusion reserve; *SRS* summed rest score; *SSS* summed stress score

* Significant *P* value

**Figure 3:**
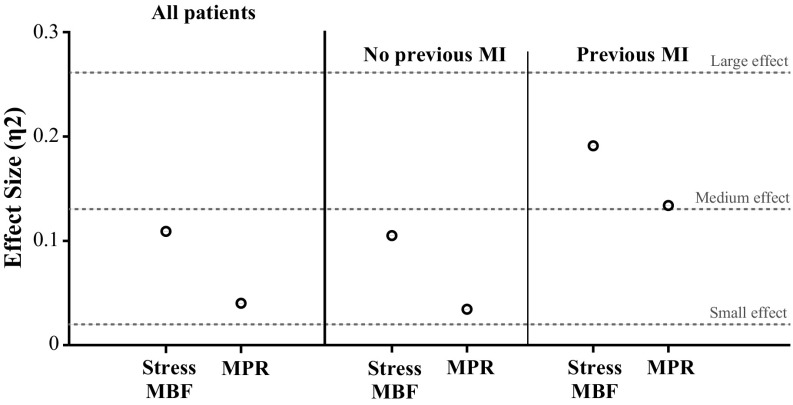
Point graph with the effect sizes of stress MBF and MPR estimated by the multivariate analyses

The secondary exploratory multivariate analysis for patients without a previous MI (n = 162) again showed that stress MBF was significantly associated with ventricular function (*P* = .006, η^2^ = 0.103), while MPR was not (*P* = .27). In this analysis, sex and BMI were also significant predictors (*P* = .029 and *P* = .005, respectively). Similar results were obtained in the analysis of patients with evidence of a previous MI: stress MBF (*P* = .022), MPR (*P* = .090), and SRS (*P* = .004). These results are shown in more detail in the Supplementary Material—Online Resources 2 and 3. The magnitude of the effect sizes for the primary and secondary analyses is depicted in Figure 3.

The supplementary analyses in women without a previous MI conveyed a rather small and, therefore, underpowered subsample (n = 65) considering the amount of relevant variables in the other analyses. As for noninfarcted patients with hypertension (n = 101), results were similar to those in the primary and secondary analyses (Supplementary Material—Online Resource 4).

A supplementary follow-up univariate analysis divided per measurement of ventricular function can be consulted in Supplementary Material—Online Resources 5 through 7.

## Discussion

In the present study, we have shown that stress MBF correlates better that MPR with ventricular function in a population of patients with suspected ischemia using an integral statistical approach. By inputting both quantitative perfusion measurements (i.e. stress MBF and MPR) in the analyses, we have aimed to compare their relative importance based on their independent association with ventricular function.

In the first part of the study, we documented differences between important subgroups concerning the presence or the absence of a previous MI. Results have confirmed important differences in ventricular perfusion and several components of ventricular function as well as in clinical profile between these two groups of patients. Patients with evidence of a previous MI had a higher prevalence of type 2 DM which adds to their prognostic profile. Interestingly, they were more symptomatic and showed slightly more ischemic burden overall. As expected, a worse ventricular function profile was found. However, not only scar but also perfusion seems to play a role in their functional status.

A close statistical relationship between systolic, diastolic, and synchronic ventricular function was observed. In the clinical scenario, functional measurements may individually serve at different points of the spectrum of severity of CAD. Nonetheless, they also provide, in compound, a proxy for the overall status of ventricular function since in reality they are probably not modified independently. Still, further research is needed to define the optimal measurements of diastolic function and synchrony, as well as their particular normal values in different clinical scenarios evaluated with PET imaging.

Next, we documented that stress MBF is consistently better correlated to ventricular function than MPR as evidenced by their significance values and moderate effect sizes in the multivariate models (Figure 3), independently from the extent and severity of a previous MI (as measured by SRS) or other clinically relevant variables. Nonetheless, it became clear in the primary analysis that the greatest influence on cardiac function patent from our analyses was the presence of a previous MI.

The significant association of stress MBF with ventricular function was sustained in the supplementary analysis of patients with hypertension and without previous MI. These results suggest a greater relevance of stress MBF, rather than MPR, in the profile of patients who may benefit from its evaluation in routine clinical practice. Moreover, considering the use of stress MBF as an alternative to MPR may be of benefit considering patient safety for the evaluation of ischemic burden. In this sense, acquisition times and radiation doses can be reduced^26^ through “stress-only” protocols. This, however, may not be implemented in patients with history of a previous MI, where rest defects should be evaluated.

We have found other significant predictors of ventricular function, although not as strong as stress MBF, namely: sex, age, and type 2 DM. These results support previous studies describing sex-based systolic function differences, a progressive decline of diastolic function associated with age,^27^ and the vascular-function-independent effect of type 2 DM on myocardial contraction.^28^

The clinical use of quantitative perfusion goes beyond diagnosis. Risk stratification and prediction of CAD-related outcomes are of major interest in noninvasive cardiac imaging due to the major burden that cardiovascular disease represents.^29^ Notably, our results support the potential utility of stress MBF over MPR in two main aspects. First, the role of stress MBF in patients that have not suffered a major event, such as MI, may be primarily diagnostic, as it has been proposed in studies focused on the detection of anatomically significant CAD.^8^ Then, stress MBF may be sensitively hampered and additionally provide information on the functional consequences of myocardial ischemia. As already mentioned, this application can potentially prevent unnecessary radiation exposure if a resting phase scan is spared. Second, in the case of patients with a previous MI, the main role of stress MBF may be prognostic,^9^,^10^ given its relevance in influencing residual ventricular function. Future research on condition-adjusted cut-offs and on the prognostic performance of stress MBF for specific outcomes, such as heart failure, is warranted.

A previous report from our group^30^ has addressed the relationship between quantitative myocardial perfusion and systolic function. However, such study was performed in a highly restricted population including only patients with previous MI and varying degrees of systolic dysfunction, and it was mostly focused on MPR rather than stress MBF. The present study extends our knowledge concerning the relationships of stress MBF by demonstrating a stronger association with ventricular function in comparison with MPR, and in a wider population of patients referred for cardiac PET imaging.

We believe that further investigation will optimize the application of cardiac PET by exploring the clinical value of combined perfusion-function measurements which may offer more integral gauges of cardiac health.

## New Knowledge Gained

This study demonstrated that peak-stress MBF correlates with ventricular function (composed by measures of systolic, diastolic, and synchronic function) better than MPR and independently from relevant covariates. Additionally, the present work showed that the described greater correlation of stress MBF with ventricular function is sustained in the patients with and without a previous MI and in noninfarcted patients with arterial hypertension.

## Limitations

One of the characteristics of our study is that we have approached a fairly heterogeneous population. Although this potentially yields difficulty in addressing multivariate relations, we believe that this approach rather provides a broad view of these complex relations in the setting of the day-to-day reality of specialized cardiovascular imaging centers. Another potential limitation of our study, especially for the assessment of rather novel diastolic function parameters such as MFR/3, is that the analysis of the gated datasets was performed with a binning of 8 frames per cardiac cycle. Although this may not be optimal, there are a number of studies which have demonstrated that the obtained assessment and derivation of the time-volume curves from 8 frames is comparable to SPECT reconstructions made using 64 frames. In the future, complementary echocardiographic evaluation can be useful. Yet, another limitation is the lack of regional perfusion analysis. Although of interest, we aimed to account for the global perfusion status and its influence on the global ventricular function. Therefore, the influence of specific regional measures cannot be evaluated from this study alone.

## Conclusions

Stress MBF is better correlated with ventricular function than MPR, as evaluated by Nitrogen-13 ammonia PET, independently from other relevant cardiovascular risk factors and clinical covariates. This relationship between coronary vasodilatory capacity and ventricular function is sustained across groups with and without a previous MI. Further research in the clinical utility of PET evaluation of stress MBF and its prognostic value is warranted.

## Electronic supplementary material

Below is the link to the electronic supplementary material.

Supplementary material 1 (DOCX 267 kb)

Supplementary material 2 (PPTX 1689 kb)

## Abbreviations

PET: Positron emission tomography
CAD: Coronary artery disease
MBF: Myocardial blood flow
MPR: Myocardial perfusion reserve
ECG: Electrocardiogram
MI: Myocardial infarction
LVEF: Left ventricular ejection fraction
MFR/3: Mean filling rate during the first third of diastolic time
