# Supplementary material for: Stress myocardial blood flow correlates with ventricular function and synchrony better than myocardial perfusion reserve: A Nitrogen-13 ammonia PET study
Source: J Nucl Cardiol. 2016 Sep 28;25(3):797–806. doi: 10.1007/s12350-016-0669-y (PMC5966471; doi:10.1007/s12350-016-0669-y)
Supplement: Supplementary file 1 — Supplementary material 1 (DOCX 267 kb) [file 12350_2016_669_MOESM1_ESM.docx]

**SUPPLEMENTARY ONLINE MATERIAL**

**Article Title:** Stress myocardial blood flow correlates with ventricular function and synchrony better than myocardial perfusion reserve: A Nitrogen-13 ammonia PET study.

**Journal:** Journal of Nuclear Cardiology

**Authors:**

Luis E Juárez-Orozco, MD

Erick Alexanderson, MD

Rudi A Dierckx, MD PhD

Hendrikus H Boersma, PharmD, PhD

Johannes L Hillege, MD PhD

Clark J Zeebregts, MD PhD

Myriam M Martínez-Aguilar, MD

Antonio Jordán-Ríos, MD

Ana G Ayala-German

Niek Prakken, MD PhD

Rene A Tio, MD PhD

Riemer H Slart, MD PhD

**Corresponding Author:**

**Erick Alexanderson, M.D.**

Chair of the Department of Nuclear Cardiology, Instituto Nacional de Cardiología “Ignacio Chávez”, Mexico City, Mexico.

E-mail: erickalexandersonmd@gmail.com

**Online Resource 1** Baseline subsamples characteristics for women and patients with arterial hypertension without a previous MI.

| **Variable** | **No previous MI**  **n=162** | **Women**  **n=65** | **Patients with Hypertension**  **n=101** | |
| --- | --- | --- | --- | --- |
| **Demographics - mean (SD)** |  |  |  | |
| Age (years) | 63.2 (11.3) | 66.7 (10.2) | 63.9 (11.2) | |
| Women/Men (n) | 65/97 | - | 44/57 | |
| BMI (kg/m^2^) | 28.0 (4.3) | 27.9 (4.2) | 28.4 (3.7) | |
| **Risk Factors - n (%)** |  |  |  | |
| Arterial hypertension | 101 (62) | 44 (68) | - | |
| Dyslipidemia | 90 (56) | 34 (52) | 65 | |
| Type 2 Diabetes Mellitus | 25 (15) | 9 (14) | 17 | |
| Smokers | 65 (40) | 21 (32) | 40 | |
| **Cardiovascular history - n (%)** |  |  |  | |
| Asymptomatic | 54 (33) | 30 (46) | 50 (50) | |
| Angina | 80 (49) | 22 (34) | 41 (41) | |
| Dyspnea | 75 (46) | 35 (54) | 40 (40) | |
| Previous Revascularization | 15 (9) | 4 (6) | 12 (12) | |
| **Semi-quantitative perfusion**  **metrics - mean (SD)** |  |  |  | |
| SRS | 1 (1) | 0 (1) | 0 (1) | |
| SSS | 5 (6) | 4 (5) | 5 (5) | |
| SDS | 5 (6) | 3 (5) | 4 (5) | |
| **Quantitative Perfusion Measurements - mean (SD)** |  |  |  | |
| Rest MBF (mL/g/min) | 0.87 (0.33) | 1.04 (0.32) | 0.91 (0.34) | |
| Stress MBF (mL/min/gr) | 2.21 (0.73) | 2.64 (0.73) | 2.23 (0.76) | |
| MPR | 2.72 (0.89) | 2.73 (0.98) | 2.63 (0.91) | |
| **Ventricular Function**  **measurements - mean (SD)** |  |  |  | |
| LVEF (systolic) | 67.9 (9.7) | 71.9 (5.8) | 68.6 (7.1) | |
| MFR/3 (diastolic) | 1.21 (0.35) | 1.16 (0.29) | 1.19 (0.32) | |
| Entropy (synchrony) | 42.3 (8.7) | 41.7 (7.5) | 41.9 (7.7) | |
| **Abbreviations:** BMI, body mass index; MI, myocardial infarction; MBF, myocardial blood flow; MPR, myocardial perfusion reserve | | | |  |

**Online Resource 2** Multivariate analysis for significant predictors of ventricular function in patients without a previous MI, n=162

| **Dependent Variables** | **Independent Variables** | **Pillai's Trace Value** | **F** | **Hypothesis df** | **Error df** | ***p-*value** |
| --- | --- | --- | --- | --- | --- | --- |
| **LVEF**  **MFR/3**  **Entropy** | Intercept | .735 | 106.6 | 3.0 | 115.0 | .000 |
|  | Sex | .075 | 3.127 |  |  | .029* |
|  | Age | .056 | 2.284 |  |  | .083 |
|  | HTN | .056 | 2.281 |  |  | .083 |
|  | Dyslipidemia | .012 | .472 |  |  | .702 |
|  | DM2 | .036 | 1.450 |  |  | .232 |
|  | Smoking | .040 | 1.582 |  |  | .198 |
|  | BMI | .107 | 4.590 |  |  | .005* |
|  | SRS | .017 | .668 |  |  | .573 |
|  | SSS | .021 | .823 |  |  | .484 |
|  | Stress MBF | .103 | 4.384 |  |  | .006* |
|  | MPR | .033 | 1.323 |  |  | .270 |

***Abbreviations:*** *BMI, body mass index; df, degrees of freedom; DM2, type 2 diabetes mellitus; E, entropy; HTN, arterial hypertension; LVEF, left ventricular ejection fraction; MBF, myocardial blood flow; MFR/3, mean filling rate during the first third of the diastole; MPR, myocardial perfusion reserve; SDS, summed difference score; *, significant p-value.*

**Online Resource 3** Multivariate analysis for significant predictors of ventricular function in patients with a previous MI, n=86

| **Dependent Variables** | **Independent Variables** | **Pillai's Trace Value** | **F** | **Hypothesis df** | **Error df** | ***p-*value** |
| --- | --- | --- | --- | --- | --- | --- |
| **LVEF**  **MFR/3**  **Entropy** | Intercept | .446 | 12.10 | 3.0 | 45.0 | .000 |
|  | Sex | .152 | 2.685 |  |  | .058 |
|  | Age | .098 | 1.628 |  |  | .196 |
|  | HTN | .008 | .125 |  |  | .945 |
|  | Dyslipidemia | .003 | .045 |  |  | .987 |
|  | DM2 | .035 | .543 |  |  | .655 |
|  | Smoking | .067 | 1.078 |  |  | .368 |
|  | BMI | .027 | .418 |  |  | .741 |
|  | SRS | .252 | 5.066 |  |  | .004* |
|  | SSS | .013 | .199 |  |  | .897 |
|  | Stress MBF | .191 | 3.542 |  |  | .022* |
|  | MPR | .133 | 2.304 |  |  | .090 |

***Abbreviations:*** *BMI, body mass index; df, degrees of freedom; DM2, type 2 diabetes mellitus; E, entropy; HTN, arterial hypertension; LVEF, left ventricular ejection fraction; MBF, myocardial blood flow; MFR/3, mean filling rate during the first third of the diastole; MPR, myocardial perfusion reserve; SDS, summed difference score; SRS, summed rest score; *, significant p-value.*

**Online Resource 4** Multivariate analysis for significant predictors of ventricular function in patients with hypertension and no evidence of a previous MI, n=101

| **Dependent Variables** | **Independent Variables** | **Pillai's Trace Value** | **F** | **Hypothesis df** | **Error df** | ***p-*value** |
| --- | --- | --- | --- | --- | --- | --- |
| **LVEF**  **MFR/3**  **Entropy** | Intercept | .729 | 71.666 | 3.0 | 80.0 | .000 |
|  | Sex | .089 | 2.617 |  |  | .057 |
|  | Age | .080 | 2.325 |  |  | .081 |
|  | Dyslipidemia | .037 | 1.019 |  |  | .389 |
|  | DM2 | .075 | 2.166 |  |  | .099 |
|  | Smoking | .084 | 2.435 |  |  | .071 |
|  | BMI | .124 | 3.791 |  |  | .013* |
|  | SRS | .011 | .290 |  |  | .833 |
|  | SSS | .031 | .867 |  |  | .462 |
|  | Stress MBF | .189 | 6.227 |  |  | .001* |
|  | MPR | .088 | 2.587 |  |  | .059 |

**Online Resource 5** Scatterplot showing the univariate follow-up regression analysis between stress MBF and the systolic function parameter

**Online Resource 6** Scatterplot showing the univariate follow-up regression analysis between stress MBF and the diastolic function parameter

**Online Resource 7** Scatterplot showing the univariate follow-up regression analysis between stress MBF and the synchrony parameter
