# Supplementary material for: Stress myocardial blood flow correlates with ventricular function and synchrony better than myocardial perfusion reserve: A Nitrogen-13 ammonia PET study
Source: J Nucl Cardiol. 2016 Sep 28;25(3):797–806. doi: 10.1007/s12350-016-0669-y (PMC5966471; doi:10.1007/s12350-016-0669-y)
Supplement: Supplementary file 2 — Supplementary material 2 (PPTX 1689 kb) [file 12350_2016_669_MOESM2_ESM.pptx]

## Slide 1
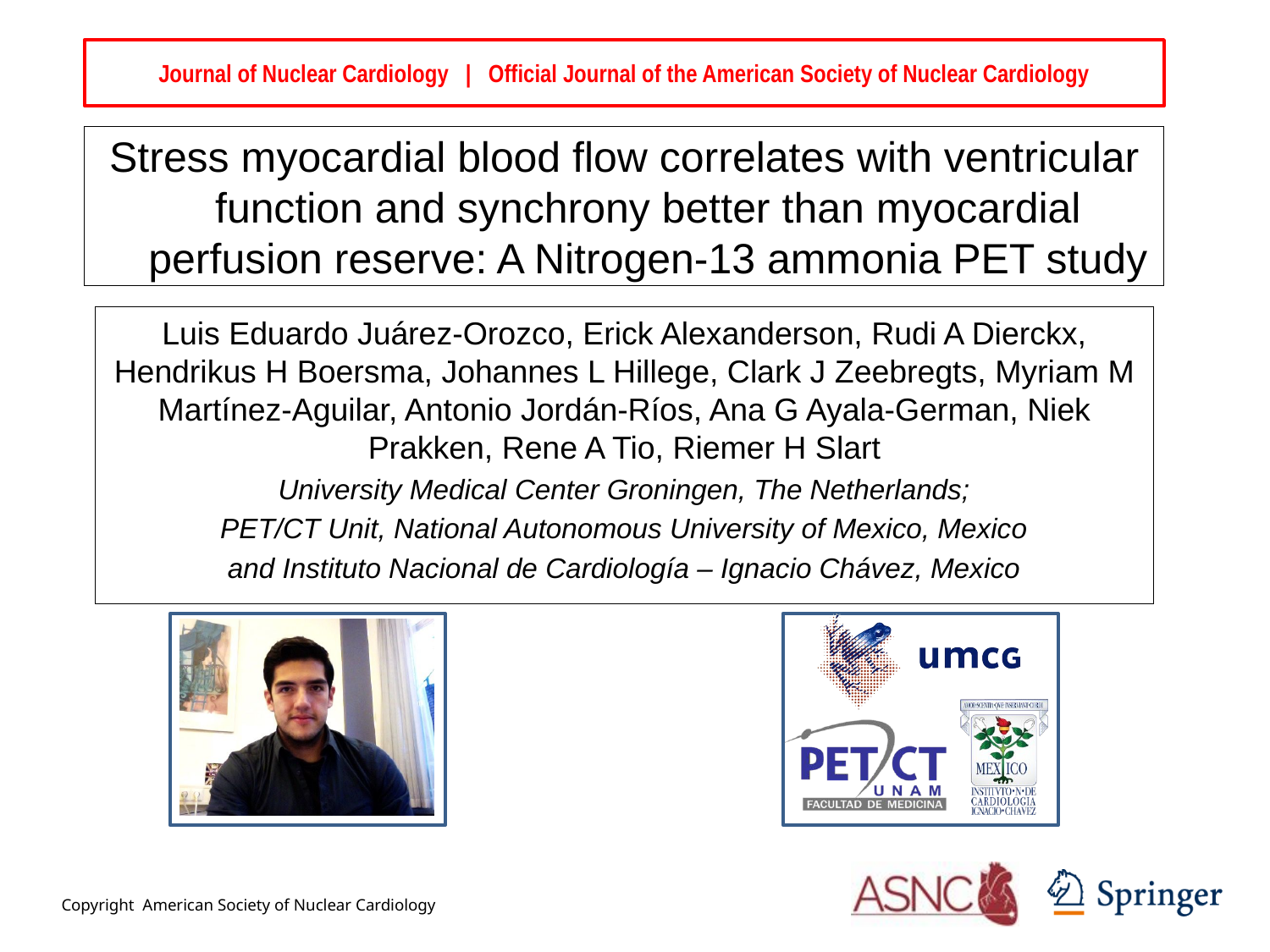

Journal of Nuclear Cardiology | Official Journal of the American Society of Nuclear Cardiology
# Stress myocardial blood flow correlates with ventricular function and synchrony better than myocardial perfusion reserve: A Nitrogen-13 ammonia PET study
Luis Eduardo Juárez-Orozco, Erick Alexanderson, Rudi A Dierckx, Hendrikus H Boersma, Johannes L Hillege, Clark J Zeebregts, Myriam M Martínez-Aguilar, Antonio Jordán-Ríos, Ana G Ayala-German, Niek Prakken, Rene A Tio, Riemer H Slart
University Medical Center Groningen, The Netherlands;
PET/CT Unit, National Autonomous University of Mexico, Mexico
and Instituto Nacional de Cardiología – Ignacio Chávez, Mexico
Head shot of author
required
Copyright American Society of Nuclear Cardiology

## Slide 2
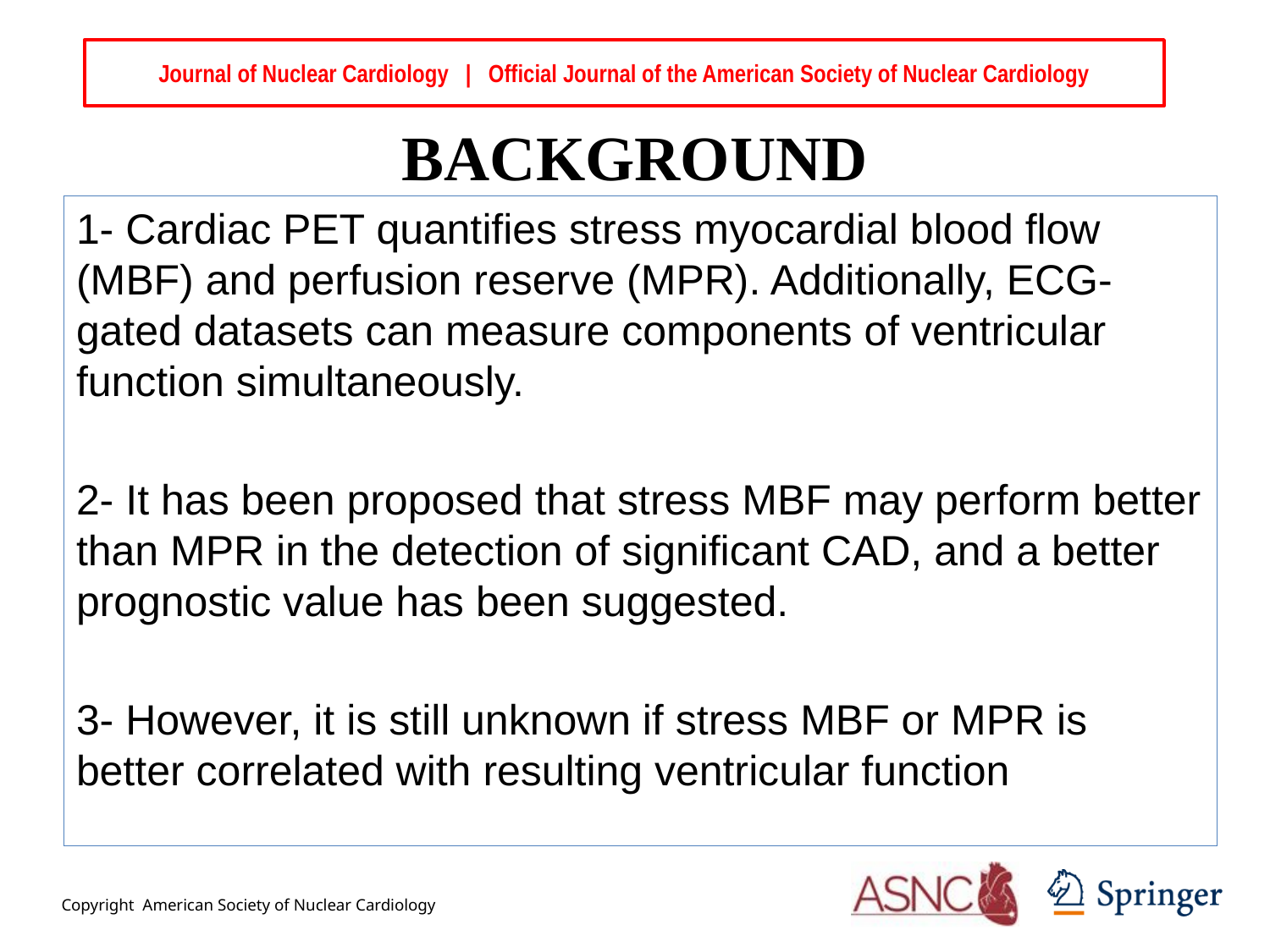

Journal of Nuclear Cardiology | Official Journal of the American Society of Nuclear Cardiology
# BACKGROUND
1- Cardiac PET quantifies stress myocardial blood flow (MBF) and perfusion reserve (MPR). Additionally, ECG-gated datasets can measure components of ventricular function simultaneously.
2- It has been proposed that stress MBF may perform better than MPR in the detection of significant CAD, and a better prognostic value has been suggested.
3- However, it is still unknown if stress MBF or MPR is better correlated with resulting ventricular function
Copyright American Society of Nuclear Cardiology

## Slide 3
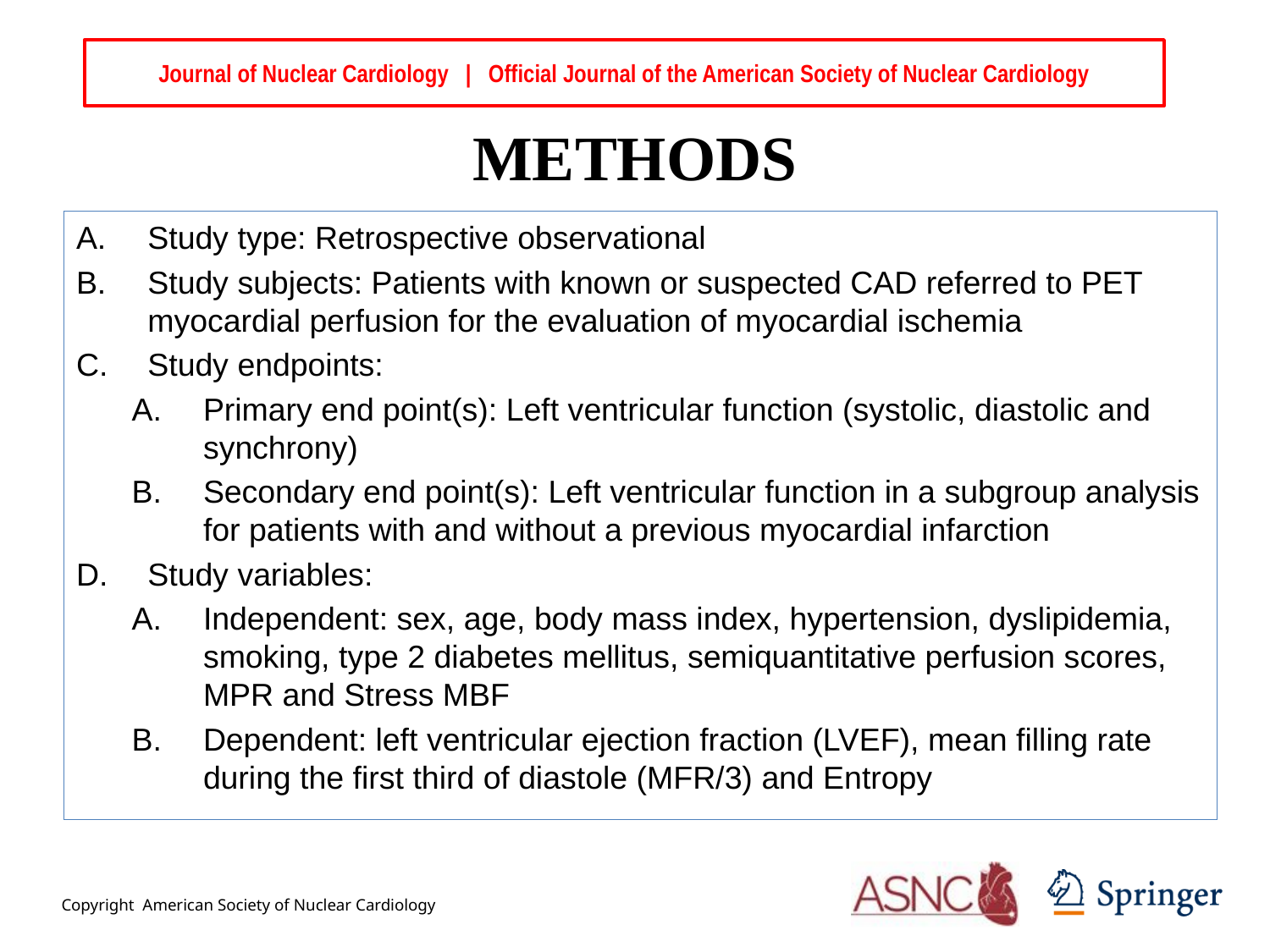

Journal of Nuclear Cardiology | Official Journal of the American Society of Nuclear Cardiology
# METHODS
Study type: Retrospective observational
Study subjects: Patients with known or suspected CAD referred to PET myocardial perfusion for the evaluation of myocardial ischemia
Study endpoints:
Primary end point(s): Left ventricular function (systolic, diastolic and synchrony)
Secondary end point(s): Left ventricular function in a subgroup analysis for patients with and without a previous myocardial infarction
Study variables:
Independent: sex, age, body mass index, hypertension, dyslipidemia, smoking, type 2 diabetes mellitus, semiquantitative perfusion scores, MPR and Stress MBF
Dependent: left ventricular ejection fraction (LVEF), mean filling rate during the first third of diastole (MFR/3) and Entropy
Copyright American Society of Nuclear Cardiology

## Slide 4
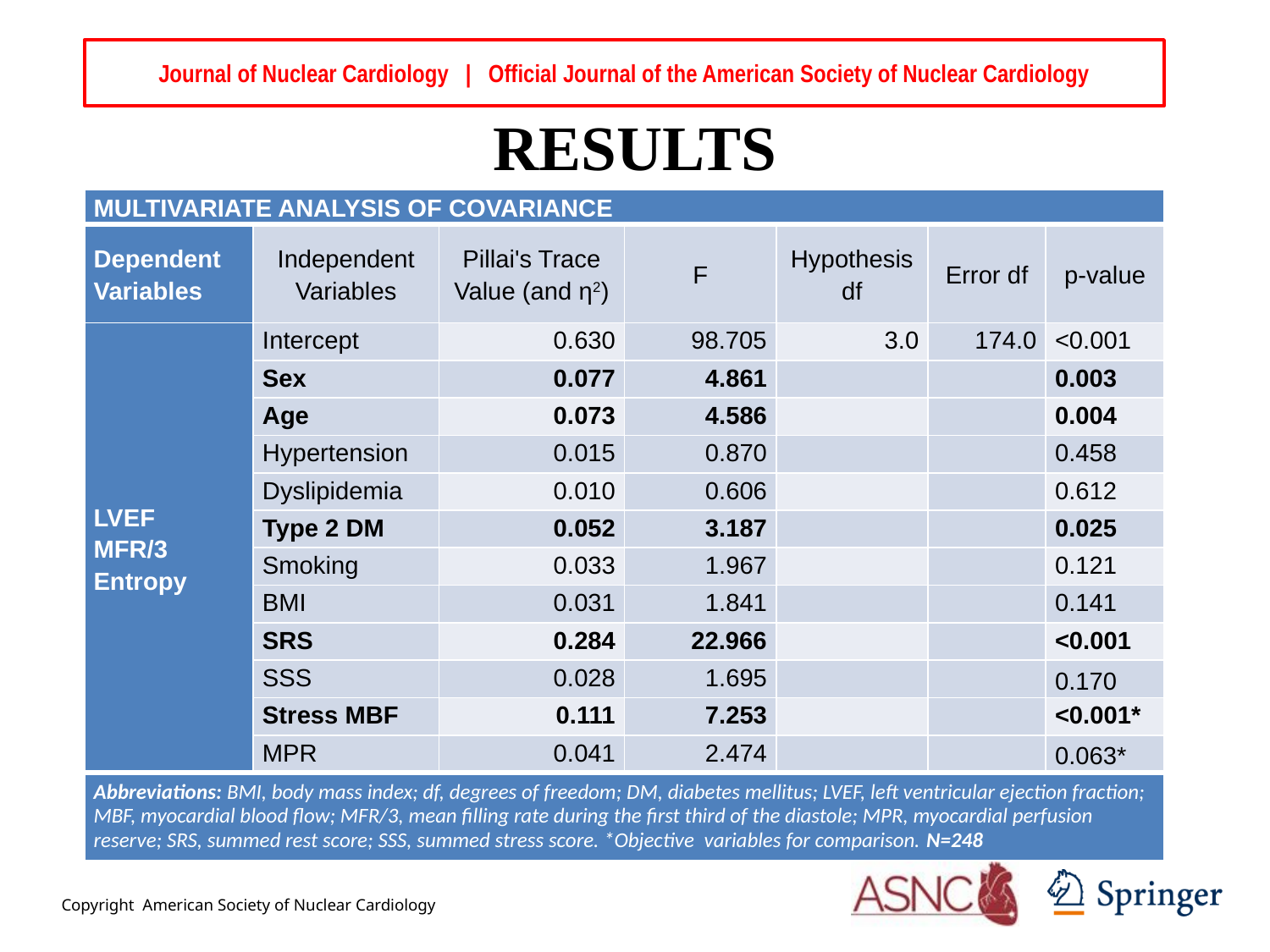

Journal of Nuclear Cardiology | Official Journal of the American Society of Nuclear Cardiology
# RESULTS
| MULTIVARIATE ANALYSIS OF COVARIANCE | | | | | | |
| --- | --- | --- | --- | --- | --- | --- |
| Dependent Variables | Independent Variables | Pillai's Trace Value (and η2) | F | Hypothesis df | Error df | p-value |
| LVEF MFR/3 Entropy | Intercept | 0.630 | 98.705 | 3.0 | 174.0 | <0.001 |
| | Sex | 0.077 | 4.861 | | | 0.003 |
| | Age | 0.073 | 4.586 | | | 0.004 |
| | Hypertension | 0.015 | 0.870 | | | 0.458 |
| | Dyslipidemia | 0.010 | 0.606 | | | 0.612 |
| | Type 2 DM | 0.052 | 3.187 | | | 0.025 |
| | Smoking | 0.033 | 1.967 | | | 0.121 |
| | BMI | 0.031 | 1.841 | | | 0.141 |
| | SRS | 0.284 | 22.966 | | | <0.001 |
| | SSS | 0.028 | 1.695 | | | 0.170 |
| | Stress MBF | 0.111 | 7.253 | | | <0.001\* |
| | MPR | 0.041 | 2.474 | | | 0.063\* |
| Abbreviations: BMI, body mass index; df, degrees of freedom; DM, diabetes mellitus; LVEF, left ventricular ejection fraction; MBF, myocardial blood flow; MFR/3, mean filling rate during the first third of the diastole; MPR, myocardial perfusion reserve; SRS, summed rest score; SSS, summed stress score. \*Objective variables for comparison. N=248 | | | | | | |
Copyright American Society of Nuclear Cardiology

## Slide 5
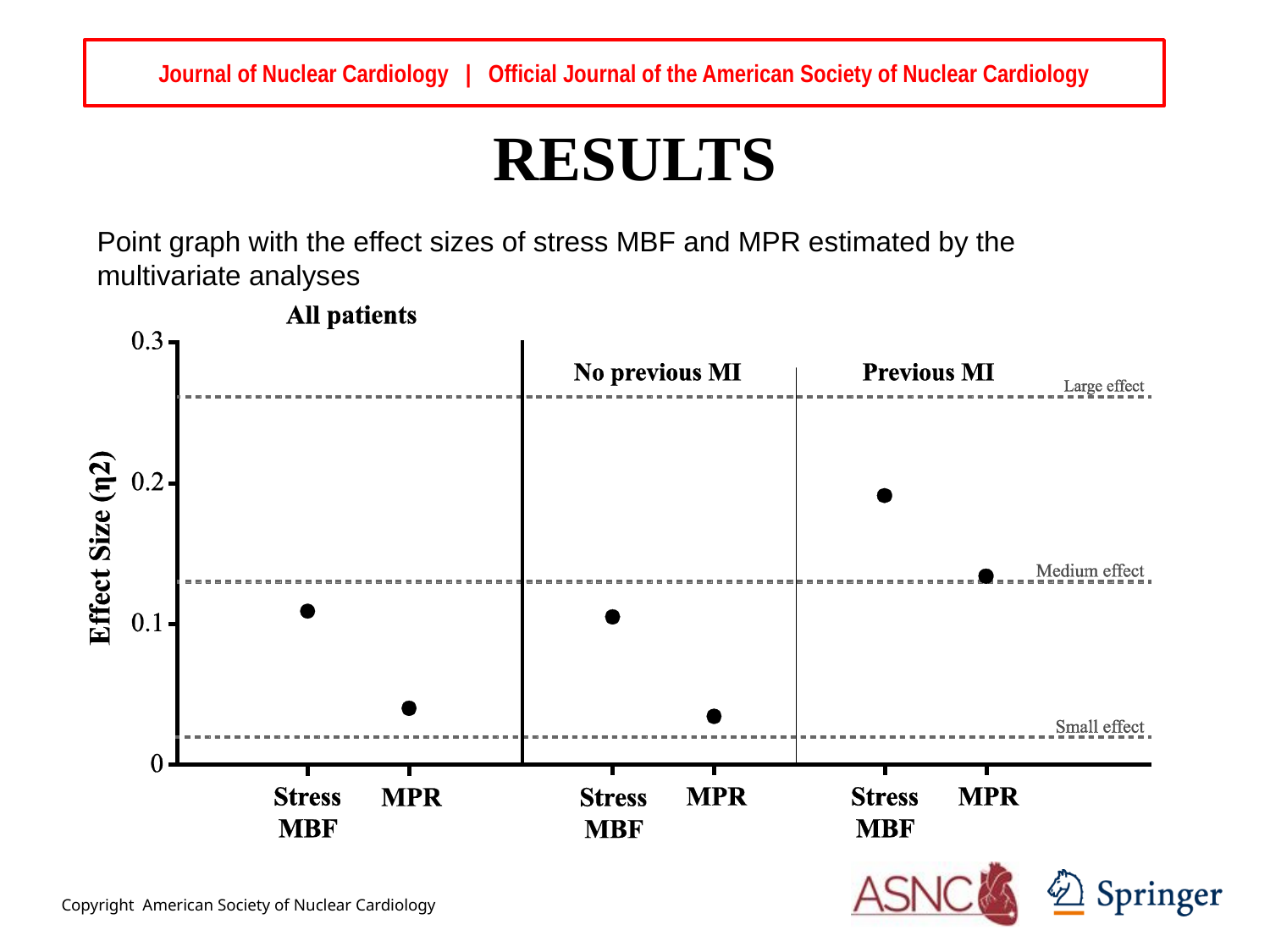

Journal of Nuclear Cardiology | Official Journal of the American Society of Nuclear Cardiology
# RESULTS
Point graph with the effect sizes of stress MBF and MPR estimated by the multivariate analyses
Copyright American Society of Nuclear Cardiology

## Slide 6
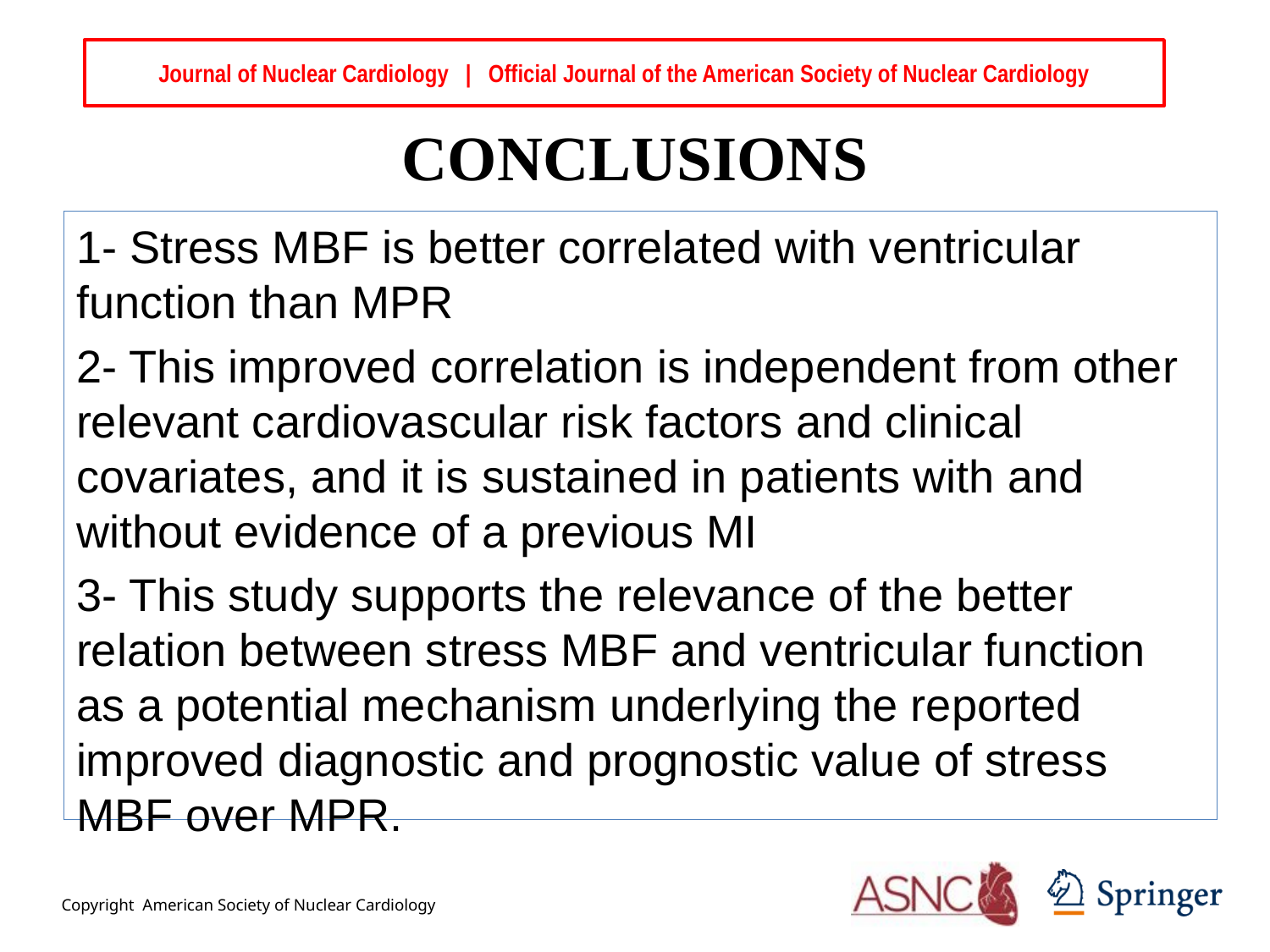

Journal of Nuclear Cardiology | Official Journal of the American Society of Nuclear Cardiology
# CONCLUSIONS
1- Stress MBF is better correlated with ventricular function than MPR
2- This improved correlation is independent from other relevant cardiovascular risk factors and clinical covariates, and it is sustained in patients with and without evidence of a previous MI
3- This study supports the relevance of the better relation between stress MBF and ventricular function as a potential mechanism underlying the reported improved diagnostic and prognostic value of stress MBF over MPR.
Copyright American Society of Nuclear Cardiology
